# Detection of Cherry Quality Using YOLOV5 Model Based on Flood Filling Algorithm

**DOI:** 10.3390/foods11081127

**Published:** 2022-04-14

**Authors:** Wei Han, Fei Jiang, Zhiyuan Zhu

**Affiliations:** 1 Chongqing Key Laboratory of Nonlinear Circuits and Intelligent Information Processing, College of Electronic and Information Engineering, Southwest University, Chongqing 400715, China; Trxiaoxin@outlook.com; 2 The 28th Research Institute of China Electronics Technology Group Corporation, Nanjing 210007, China; jiangfei_nj@163.com

**Keywords:** flood filling algorithm, deep learning, image processing, YOLOv5s, cherry

## Abstract

Presently, the quality of cherries in the market is uneven, because human senses are used to distinguish cherry quality, which consumes a lot of time and energy and does not achieve good results in terms of accuracy. If the internal quality indices, such as the PH value, sugar–acid ratio, and vitamin C content, of cherries are extracted using chemical methods, the detection speed will decrease. With the development of artificial intelligence (AI), image processing by AI algorithms has attracted broad attention. The YOLOv5 model in the YOLO series has many advantages, such as high detection accuracy, fast speed, small size, and so on, and has been used in face recognition, image recognition and other fields. However, owing to the influence of seasonal weather, the environment and other factors, the dataset used in the training model decreases the accuracy of image recognition. To improve the accuracy, a large amount of data must be used for model training, but this will decrease the model training speed. Because it is impossible to use all data in training, there will inevitably be recognition errors in the detection process. In this study, the cherry images in a dataset were extracted by the flooding filling algorithm. The extracted cherry images were used as a new dataset for training and recognition, and the results were compared to those obtained with non-extracted images. The dataset generated by the flooding filling algorithm was used for model training. After 20 training epochs, the accuracy rate reached 99.6%. Without using the algorithm to extract images, the accuracy rate was only 78.6% after 300 training epochs.

## 1. Introduction

Cherries are distributed in the United States, Europe, Australia and other countries. In China, cherry farming originated in the Yangtze River Basin, particularly in Zhejiang, Shandong, Henan, Jiangsu and other areas. Cherries are rich in nutrients and loved by people. The quality of the fruit determines the market competitiveness. The quality of the cherry is the result of the combined effect of genes and environmental factors. The evaluation of its quality includes external and internal indicators. However, because cherries are non-storage fruit and harvested in midsummer, their quality easily declines owing to environmental factors, such as the temperature during storage, transportation, and shelf life, which affect sales [1,2,3,4,5,6]. It is specifically reflected in the changes of fruit stalk length, transverse diameter, longitudinal diameter, fruit shape, color, texture characteristics and chemical composition. Computer vision technology can quickly, accurately and non-destructively detect and evaluate external features, such as color, texture and surface defects of cherries, and then determine the variety and classification level of cherries. In the literature [7], five planted large cherry varieties were used as materials to discuss the significance of differences in fruit appearance and endoplasmic indicators. Although measuring 12 quality indicators, such as single fruit quality, transverse diameter, longitudinal diameter, sugar–acid ratio, and vitamins, through traditional chemical methods can accurately and comprehensively evaluate the quality of cherries, each item of data needs to be manually tested, meaning it will consume a lot of time and energy. The detection effect of shelf life is not ideal. In the literature [8,9], correlation analysis was carried out on various indicators of cherries, and factor analysis was used to extract main factors and simplify fruit evaluation indicators, but the improvement in detection speed and portability was not obvious. With the development of artificial intelligence technology, convolution neural networks (CNNs) can extract and classify image features and directly identify the original image. Moreover, CNNs have been successfully used for face recognition [10] and license plate recognition [11], and achieved good results. Zeng et al. [12] constructed a CNN with two convolution layers, two pooling layers, and two fully connected layers. By extracting features from the bottom layer and further extracting deeper features, apples, pears, oranges, and peaches were identified, with an accuracy of 98.44%, and the cumbersome process of manually extracting features was avoided. Huang et al. [13] studied a fruit image algorithm based on multi-scale feature fusion to investigate the missing and false detection phenomenon in the process of fruit recognition. By using ResNet-50 as the main stem network, they expanded the fruit image data in Fruits-360, further normalized the gray level, and added a batch normalization layer after each convolution layer. Based on the original features, they obtained a feature map with more abundant information, and the final recognition accuracy reached up to 99.4%, which is superior to that of other mainstream algorithms with the same data. However, the training still required 150 iterations to stabilize the accuracy and loss rate. Liu et al. [14] designed a fruit recognition system based on TensorFlow. More than 70 types of fruit data on Kaggle and approximately 30,000 pictures were used. The dataset was used to train a CNN and all fruit features were obtained. The accuracy rate reached 100% and the training required 5000 iterations, which has certain practical value. Luo et al. [15] designed a fruit recognition system based on a back propagation (BP) neural network. By considering apples, oranges, and bananas as the objects, the texture, shape, and color features were extracted and used as the training input for the BP neural network. After testing, the success rate of recognition reached 93.18%. Sun et al. [16] proposed a recognition algorithm based on optimized particle swarm optimization combined with a BP neural network. Although the image segmentation efficiency and recognition ability of their algorithm is not as good as deep learning, a superior fruit recognition rate was achieved after optimization and the time of the overall algorithm was effectively controlled, which is valuable for improving the accuracy of fruit recognition.

Lei et al. [17] introduced the detection principle of near-infrared spectroscopy, reviewed the research progress of infrared spectroscopy in fruit quality detection, and investigated the application of infrared spectroscopy in fruit quality detection. Using near-infrared spectroscopy, Luo et al. [18,19] established a qualitative identification model of the cherry fruit quality during storage, based on different pretreatments and spectral band conditions. The final accuracy rate reached 88.9–99%, and the rapid evaluation of internal fruit quality was realized.

To further improve the recognition accuracy and model generation speed, this study used images optimized by the flooding filling algorithm as the training and test datasets, and also used the YOLOv5s network with the smallest depth and smallest width of the feature map in the YOLOv5 series for training. The influence of environmental factors on the training speed and recognition effect of the model was eliminated. Compared with previous cherry quality recognition methods, the training time improved, only 20 iterations were required, and the recognition accuracy reached 99.6%. Finally, the model generation speed and recognition accuracy were greatly optimized. At the same time, the algorithm can be embedded in a mobile application and the mobile application will allow the image taken with the phone camera to be used as input to the algorithm, which will make it easy for users to obtain the quality information of a cherry after taking photos from a mobile device.

## 2. Model and Algorithm

### 2.1. Convolutional Neural Network

**Figure 1:**
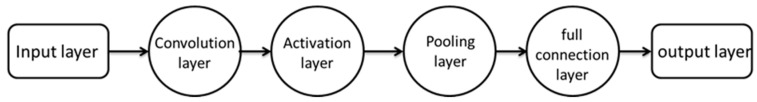
Basic structure of CNN.

The CNN can recognize the original image by feature extraction. The original CNN mainly consists of an input layer, convolution layer, activation layer, pooling layer, and full connection layer, and is a feedforward neural network. The basic model structure is shown in Figure 1.

Compared with traditional deep neural networks (DNNs), CNNs are different in terms of the convolution layer and pooling layer. The activation function of the convolution layer uses the Relu function, which is expressed as f(x)=max(0,x). The convolution layer is the core structure of the CNN network and can reduce the dimension of high-dimensional input image data through the convolution kernel and extract excellent image features. The principle of the CNN is that the convolution kernel moves continuously on the input image data of the input layer, and the convolution operation is carried out simultaneously. Generally, image data are multi-dimensional. Compared with the convolution layer, the pooling layer is relatively simple and typically located behind the convolution layer, and its function is to extract the local mean and maximum values. According to the different calculated values, the pooling layer is divided into the mean pooling layer and maximum pooling layer, and the pooling layer does not have the same activation function as the convolution layer. The combination of the convolution layer and pooling layer can occur many times in the hidden layer and can be flexibly combined, according to the actual needs of model construction, such as by combining two convolution layers, or combining two convolution layers and a pooling layer. In model construction, these combinations are unlimited, and the most common combination is the combination of several convolution layers and pooling layers.

### 2.2. Flooding Filling Algorithm

The flooding filling algorithm is a common filling algorithm. The principle of the algorithm is to begin from a starting node and extract or fill the nearby connected nodes in different colors until all nodes in the closed region are processed. The classic algorithm extracts several connected nodes from a region and distinguishes them from other adjacent regions. The algorithm receives three parameters: the starting node, the characteristics of the target node, and the processing to be performed on the extracted object. The algorithm is typically used in the four neighborhoods filling method, eight neighborhoods filling method, and scanning line filling method.

In this experiment, the flooding filling type algorithm was used. The algorithm started from the edge of each image and marked all pixels there, and then marked all pixels whose distance between the colors found in the neighborhood of the marked pixels was less than the specified value. This step was repeated until no more pixels could be marked. All marked pixels were considered to be the background filled with white color, and unlabeled pixels were considered to belong to the object. The maximum distance between two adjacent pixels is a parameter of the algorithm

### 2.3. YOLOv5 Model

This study used the YOLOv5s model for training and learning. 

**Figure 2:**
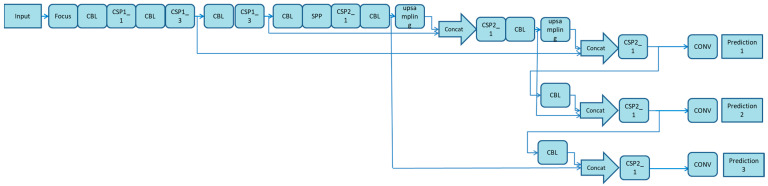
Network structure diagram of YOLOv5s target detection algorithm.

As shown in Figure 2, the YOLOv5s network can be divided into four parts: the input, backbone network, neck and output. The input uses the same mosaic data enhancement method as YOLOv4. For different datasets, there will be an anchor box with an initial set width. In network training, the network outputs a prediction box based on the initial anchor box, then compares it with the actual box to calculate the gap, and finally, reversely updates and iterates the network parameters. When training different datasets with YOLOv3 and YOLOv4, the initial anchor frame value passes through a separate program. However, this function is embedded into the YOLOV5 code. During each training epoch, the optimal anchor frame value in different training sets is adaptively calculated. In the commonly used target detection algorithm, the length and width of different images are different. A common method is to uniformly scale the original image to a standard size and then send it to the detection network. However, owing to the different aspect ratios of many images, after scaling and filling, the size of the black edges at both ends is also different. If the filling is excessive, it will cause information redundancy and, thus, affect the reasoning speed. In YOLOv5, the least black edges are adaptively added to the original image. In the reasoning, the calculation amount will also be reduced, and the target detection speed will greatly improve. In essence, the backbone network is a CNN that can extract image characteristics. Notably, YOLOv4 only uses the constraint satisfaction problems (CSP) structure in the backbone network, and there is no focus structure. In YOLOv5, the focus structure uses 32 convolution cores. There are two CSP structures used in the backbone network and neck. The combination of the Feature Pyramid Network (FPN) and Pixel Aggregation Network (PAN) feature pyramids is used in the neck, but the positioning information is not transmitted. The YOLOv5 model enhances the image preprocessing through mosaic data. In the neck structure of YOLOV4, an ordinary convolution operation is used. In the neck structure of YOLOv5, the CSP2 structure designed by CSP net is used to enhance the network feature fusion ability.

## 3. Experiment and Results

### 3.1. Data Set Preparation

In this experiment, the dataset produced with the original image and that produced after the image was extracted by the flooding filling algorithm were used for training, respectively, and the results were compared. We divided four different qualities of cherries for a total of 400 sample images. We divided cherries into four grades by external quality indicators, such as peel color, single fruit quality, freshness, maturity, fruit shape, and fruit surface.

**Figure 3:**
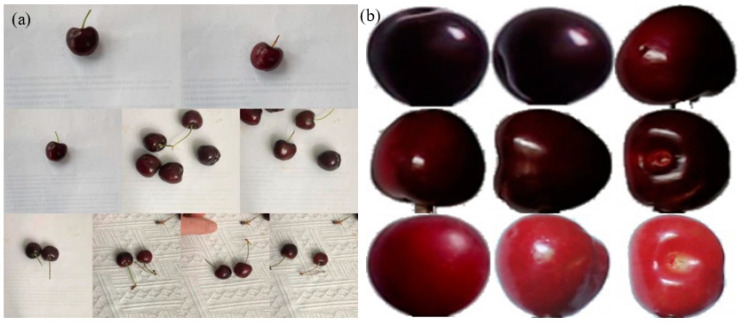
Sample images: (**a**) original sample images; (**b**) images extracted by flooding filling algorithm [20].

In order to enrich the training samples to improve the model performance, we rotated the images 90 degrees, 180 degrees, 270 degrees and flipped the images. Then, the flooding filling algorithm extracted the cherry image from the background, and 2000 datasets were produced. Following this, the cherry image was labeled by labeling software, called labelimg, and an XML document was generated. Subsequently, the image was transformed into the data format required for YOLOv5 training. Four-fifths of the image data was used as training data and one-fifth was used as test data. The cherry dataset comes from the literature [20]. Some sample images are shown in Figure 3.

### 3.2. Experimental Settings

As shown in Table 1, this experiment was deployed on the windows 10 operating system, and the YOLOv5 environment was built using the Pytorch framework. The specific hardware, software, and parameters are set according to [21].

### 3.3. Results

**Figure 4:**
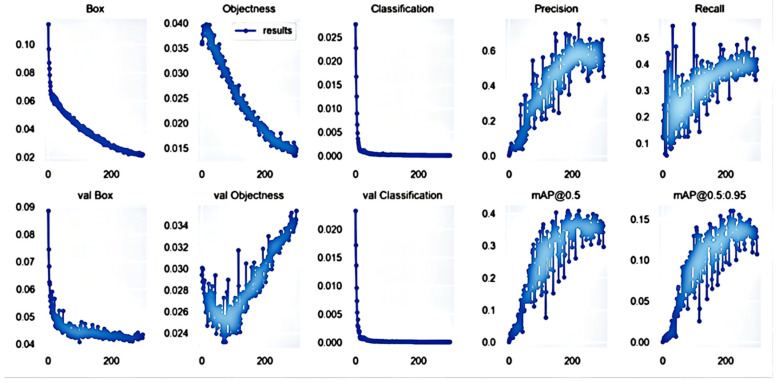
Training results obtained with original image.

**Figure 5:**
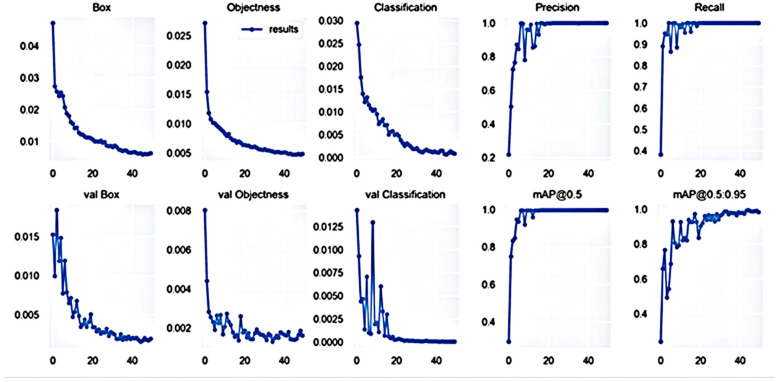
Training results obtained with image dataset extracted by flooding filling algorithm.

The original image and the dataset extracted from the background by the pan-red filling algorithm were used for training, respectively, and the precision, recall, and average precision were used as indicators to assess the target detection algorithm. Precision refers to the proportion of the correct identification of cherry quality in all identified images. The recall rate refers to the proportion of correctly identified cherries in the total dataset. The test result parameters are listed in Figure 4 and Figure 5.

As shown in Figure 4 and Figure 5, the accuracy of the trained model after image extraction reached 99.6% after 20 epochs. The accuracy of the model without image extraction was only 78% after 300 epochs. The specific comparison results are listed in Table 2.

## 4. Discussion

**Figure 6:**
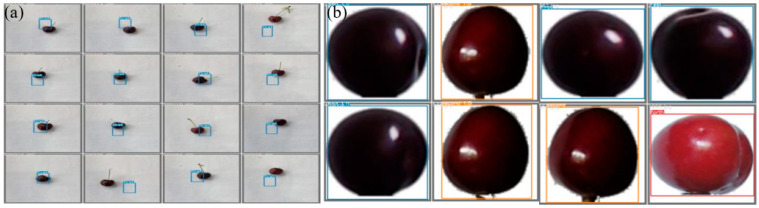
Detection effect: (**a**) detection effect with original image; (**b**) detection effect with image extraction using flood filling algorithm [20].

From the training results obtained with two different datasets, it can be seen that, after 300 training epochs, the accuracy rate of the dataset produced by the original image was only 78%, and many images have identification errors or were not identified. Moreover, the improvement of the accuracy rate is not obvious when the number of training rounds continues to increase. In the case of the same number of datasets, the image dataset extracted by the flooding filling algorithm was used for training over 50 epochs, and the accuracy rate reached 99.6% in the 20th epoch. In contrast, the training speed and recognition effect greatly improved. Some test results are shown in Figure 6.

Compared with the CNN [12], the dataset used in this experiment eliminates the influence of different seasons and environments by using the flooding filling algorithm. Thus, it is no longer necessary to iterate 150 times or even thousands of times in the process of model training to ensure a high recognition rate. In this experiment, a total of 2000 datasets with four different cherry qualities were used, and each cherry quality dataset contained 500 images. Compared with the BP neural network dataset [15], the number of samples was effectively reduced, the sample feature extraction improved, the influence of environmental factors was eliminated, YOLOv5s could effectively extract the cherry image characteristics within a short period of time, and the training accuracy reached a high level. Thus, it is demonstrated that the dataset obtained after preprocessing the original image using the flooding filling algorithm is more conducive to the model’s training and learning.

Compared with near-infrared diffuse reflection technology [22,23], near-infrared spectroscopy is one of the most promising potential analytical tools in this field. It plays a very good role in both qualitative [24] and quantitative [25] analysis, but it requires high-level hardware. The advantages of computer vision technology lie in detection speed and portability, and the algorithms can be embedded in mobile applications; the quality information of cherries can be obtained in a short time by taking corresponding images through mobile devices. The proposed method simplifies the cumbersome process of cherry quality detection, greatly improves the detection speed, and achieves certain improvement in terms of accuracy.

Although the images used in this experiment were preprocessed by the flooding filling algorithm, which greatly improved the training speed of the model, reduced the number of iterations, and improved the accuracy rate, various shortcomings still exist. Because the training dataset consisted of preprocessed images, the test images used in the identification of cherry quality also need to be preprocessed. Moreover, because only four different quality cherry images were used in model training, the datasets were not sufficiently rich and cherries with different qualities that had not appeared in training could not be identified. In future experiments, the practicality of the model can be improved by improving the richness of sample types and real-time detection. Due to the focus on improving the detection speed and accuracy, the internal quality indicators, such as sugar–acid ratio, pH value, vitamins and other internal quality indicators of cherries, cannot be included in the scope of investigation, which has certain limitations. We may try to build a DNN model in the future. Quality indicators [26] are included in the scope of investigation or try to create a database of the mapping relationship between cherry varieties and their internal components. While improving the detection speed and practicability, the discrimination results are more accurate and scientific.

## 5. Conclusions and Shortcomings

This study combined the flooding filling algorithm with the YOLOv5 target detection algorithm. The dataset required for training the YOLOv5 model was optimized by the flooding filling algorithm, which effectively reduced the impact of environmental factors on the training of the YOLOv5 model. The training results were compared to those obtained with the original data without optimization by the flooding filling algorithm. The results reveal that the training accuracy of the original image processed by the flooding filling algorithm is significantly higher (up to 99.6%) compared with that of the original image, when the same datasets were used for training and the same number of iterations was required. Specifically, only 20 iterations were required to ensure high accuracy, which significantly improved the model’s generation speed. In future work, improvements can be made with regard to the following aspects. First, the existing datasets should be expanded to increase the sample diversity. Second, the algorithm used in the model should be optimized, and the feature extraction and weight optimization should be strengthened to further improve the generation speed and accuracy of the model. We will also try to improve the mobile application for judging the quality of cherries in future work.

## Figures and Tables

**Table 1:** Experimental Configuration and Parameters.

Configuration		Parameters
Software	System	Windows 10
	IDEL	Pycharm
	Python	Python 3.8
Hardware	CPU	Intel(R) Core(TM) i7-8750H CPU @ 2.20 GHz
	Graphics card	NVIDIA GeForce GTX 1050 Ti
Training parameters	Pre-training weight	YOLOv5s.PT
	Epochs	50
	Sample size	2000

**Table 2:** Comparison of Training Results Obtained with Different Datasets.

Dataset	Precision	Recall	Average Precision (IoU Threshold: 0.5)	Average Precision (IoU Threshold: 0.5–0.95)
Original images	99.6%	99.4%	96.7%	95.4%
Images extracted by flooding filling algorithm	78.6%	58.7%	41.5%	17.8%

## Data Availability

Data available on request due to restrictions of privacy.
